# A systematic review comparing neurodevelopmental outcome in term infants with hypoxic and vascular brain injury with and without seizures

**DOI:** 10.1186/s12887-018-1116-9

**Published:** 2018-05-02

**Authors:** T. R. De Haan, J. Langeslag, J. H. van der Lee, A. H. van Kaam

**Affiliations:** 1 Department of Neonatology, Emma Children’s Hospital, Academic Medical Center, PO Box 22660, 1100 DD Amsterdam, the Netherlands; 2 Pediatric clinical Research Office, Emma Children’s Hospital, Academic Medical Center, Amsterdam, The Netherlands

## Abstract

**Background:**

There is increasing evidence that neonatal seizures in term neonates with stroke, asphyxia or brain haemorrhage might be associated with adverse neurodevelopment and development of epilepsy. The extent of this association is not known. The objective of this study was to assess the possible impact of neonatal seizures on these outcomes and if possible calculate a relative risk.

**Methods:**

A systematic review and meta-analysis was performed (study period January 2000–June 2015). PubMed, Medline and Embase were searched for cohort studies evaluating neurodevelopmental outcome at the age of at least 18 months or development of epilepsy in surviving term neonates with or without neonatal seizures. The methodological quality of included studies was assessed and data extractions were performed in a standardized manner by independent reviewers. Pooled Relative Risks (RR) with 95% confidence intervals for adverse outcome were calculated if possible.

**Results:**

Out of 1443 eligible studies 48 were selected for full text reading leaving 9 cohort studies for the final analyses (4 studies on stroke, 4 on perinatal asphyxia and one on cerebral hemorrhage). For all cases with stroke or asphyxia combined the pooled risk ratio (RR) for adverse outcome when suffering neonatal seizures was 7.42 (3.84–14.34); for neonates with perinatal asphyxia: 8.41 (4.07–17.39) and for neonates with stroke: 4.95 (1.07–23.0). The pooled RR for development of late onset epilepsy could only be determined for infants suffering from stroke: 1.48 (0.82–2.68). Results were biased and evidence sparse.

**Conclusions:**

The presence of neonatal seizures in term newborns with vascular or hypoxic brain injury may have an impact on or be a predictor of neurodevelopmental outcome. The biased available data yield insufficient evidence about the true size of this association.

**Electronic supplementary material:**

The online version of this article (10.1186/s12887-018-1116-9) contains supplementary material, which is available to authorized users.

## Background

Neonatal seizures are frequently encountered with a reported yearly incidence of 1–3 per 1000 live births [1]. Neonatal seizures confront clinicians with many diagnostic and therapeutic challenges in the neonatal intensive care unit (NICU) [2]. The developing brain is highly vulnerable to hypoxic–ischemic insults and these insults therefore often result in neonatal seizures [3, 4].

It has been hypothesized that this hyper excitability of the brain is caused by interplay between a high density synaptic network, an abundance of glutaminergic neurons, paradoxical neonatal excitatory actions of primary inhibitory networks and differences in membrane potentials in the immature dendrites [1, 3, 5].

The most important causes of neonatal seizures are hypoxic ischemic encephalopathy (HIE) due to perinatal asphyxia (50–60%), stroke (10%), and cerebral haemorrhage (10%) [3, 6].

There is increasing evidence that neonatal seizures are associated with adverse neurodevelopmental outcome, defined as cerebral palsy, psychomotor retardation and the development of post-neonatal epilepsy [2, 4, 7–9]. This may be caused by the fact that neonatal seizures add damage to an already injured brain [3, 10] and to the fact that neonatal seizures alter neuronal circuitry in the developing brain leading to an enhanced susceptibility to post-neonatal epilepsy [1].

This study’s objective was to determine if the presence of seizures in newborns with a perinatal insult to the brain (either hypoxia-asphyxia, stroke or hemorrhage) is associated with a worse long term outcome and increased risk for epilepsy in survivors. To answer this question a systematic review on this topic, including a meta-analysis were performed.

## Methods

We followed the guidelines from the Meta-analysis of Observational Studies in Epidemiology (MOOSE) [11], Preferred Reporting Items for Systematic Reviews and Meta-Analyses the (PRISMA) statements [12], and the Cochrane Handbook for Systematic Reviews of Diagnostic Test Accuracy. For this study none of the authors had any competing interests.

### Search strategy

We searched the bibliographical databases PubMed, Medline and Embase for cohort studies evaluating the long term neurodevelopmental outcome and later development of epilepsy in term newborns with hypoxic or vascular brain injury suffering from neonatal seizures.

The study period ranged from January 2000 - up until June 2015 and the search syntax can be found in Additional file 1. Older literature was not searched for as the standard of care in neonatal units has developed strongly in the last decade. Citations of eligible studies were checked for additional articles that might also be included.

### Study selection/eligibility criteria

Studies were included if they: 1) reported on the presence of neonatal seizures in term (GA > 37 weeks) infants; 2) excluded congenital brain abnormalities; 3) reported the etiology of the observed neonatal seizures; 4) the method of seizure diagnosis was described and consisted of at least a neurophysiology diagnosis either by full lead EEG or aEEG.; 5) reported on neurodevelopmental outcome of survivors with a minimum follow up age of 18 months using outcome and age appropriate neurodevelopmental testing or neurological examination, or on the presence or absence of post neonatal epilepsy at follow-up visits at any time. Both prospective and retrospective studies were eligible for inclusion.

Adverse outcome in survivors had to be defined by one or more of the following criteria: 1) the presence of cerebral palsy, either diagnosed by standardized clinical neurological examination or scored by the GMFCS - gross motor functional classification system; 2) a test score of at least 2 standard deviations below the reference mean on the Bayley Scales of Infant Development (BSID) or the Griffith Mental Developmental Index or any other age appropriate neurodevelopmental test.

The presence of post neonatal epilepsy in survivors had to be clearly documented by full lead EEG diagnosis with either the need for continued antiepileptic drug (AED) use or the need for continued pediatric neurology outpatient visits. Studies only reporting clinical suspicion of seizures were not included for analysis.

Studies were excluded if: 1) they reported only experimental data; 2) they were performed in preterm infants; 3) they only reported clinical suspicion of seizures without neurophysiological diagnostics; 4) they reported on rare (either metabolic or other) syndromes; 5) they did not report developmental outcome data at > 18 months of age or were not published in English. Also studies where it was not possible to extract data (e.g. original data not (clearly) described) or where it was not possible to make two by two tables were excluded from the analyses.

From the literature search a study selection was primarily made on the basis of Title, Abstract, Date and language. Full text articles were retrieved for final assessment. The same two reviewers who made the selection appraised the methodological quality (JL, TRH) and performed the data extraction independently. Disagreements were resolved by discussion until consensus was reached.

Studies were only included for further analyses if each major category (see also Additional file 2: patient selection; diagnosis of neonatal epilepsy; reference standard outcome and flow and timing) could be assessed.

### Methodological quality/critical appraisal

Due to a lack of existing quality assessment tools for prognostic studies, we developed a tool based on the modified version of the QUADAS-2 assessment tool [13] to evaluate the methodological quality of included studies. This tool (see also Additional file 2) consisted of 4 main items to assess methodological quality: 1) the patient selection process; 2) diagnosis of main determinant (neonatal seizures); 3) independent assessment of the outcome and 4) flow and timing.

Each main item was scored either positive or negative for good methodological quality by assessing sub-items answering questions regarding patient selection; inappropriate exclusion; sample properties; bias; blinding; conduct and interpretation of diagnostic testing and main outcome; uniformity in outcome assessment and percentage of loss to follow –up (if known). The number of items to be scored with yes/no/unclear was 20.

### Data-extraction

Data extraction was performed using a standardized data extraction form (Additional file 3). As etiology is thought to influence prognosis to a great extent, extracted data were first categorized into different etiological subgroups: HIE, stroke and cerebral hemorrhage. Next, these subgroups were categorized as newborns with or without neonatal seizures, defined as seizures in the first 28 days of life. Extracted data were entered in 2 by 2 tables.

Separate 2 by 2 tables were made for the 2 outcomes of interest; 1) adverse or normal neurodevelopmental outcome and 2) post neonatal epilepsy present or absent.

Further data extracted were: year of publication, first author, disease (stroke; asphyxia or hemorrhage), mode of seizure diagnosis, imaging modality, mortality, total number of newborns in cohort, number of newborns with neonatal seizures, number of newborns with adverse outcome or with favorable outcome, number of newborns with post-neonatal epilepsy, study-design: prospective or retrospective; single or multicenter.

### Data analysis

All data were entered in Review Manager version 5.3. Copenhagen: The Nordic Cochrane Centre, The Cochrane Collaboration, 2012; http://community.cochrane.org/help/tools-and-software/revman-5/revman-5-download). To calculate a pooled Relative Risk with 95% confidence interval (CI) for adverse outcome or development of postnatal epilepsy in patients with or without neonatal seizures we performed a meta-analysis using a univariate modeling approach. In view of the observed heterogeneity, a random-effects model was used. Heterogeneity was explored by the test for heterogeneity (I^2^), which can be interpreted as the proportion of total variation observed between the trials attributable to differences between the studies, rather than chance. An I^2^ of more than 40% was considered to represent a heterogeneous meta-analysis.

## Results

### Eligible studies

Our primary search yielded 1443 studies, of which 1387 did not fulfill inclusion criteria, three studies were not written in English and 5 were narrative reviews with theoretical background information on this subject.

A total of 48 articles were selected for full text reading. From these 48 articles 39 studies were excluded as data could not be reliably extracted, leaving 9 studies for the final analyses: 4 studies on stroke, [14–17], 4 studies on HIE, [18–21] and one study on cerebral hemorrhage [22].

**Fig. 1:**
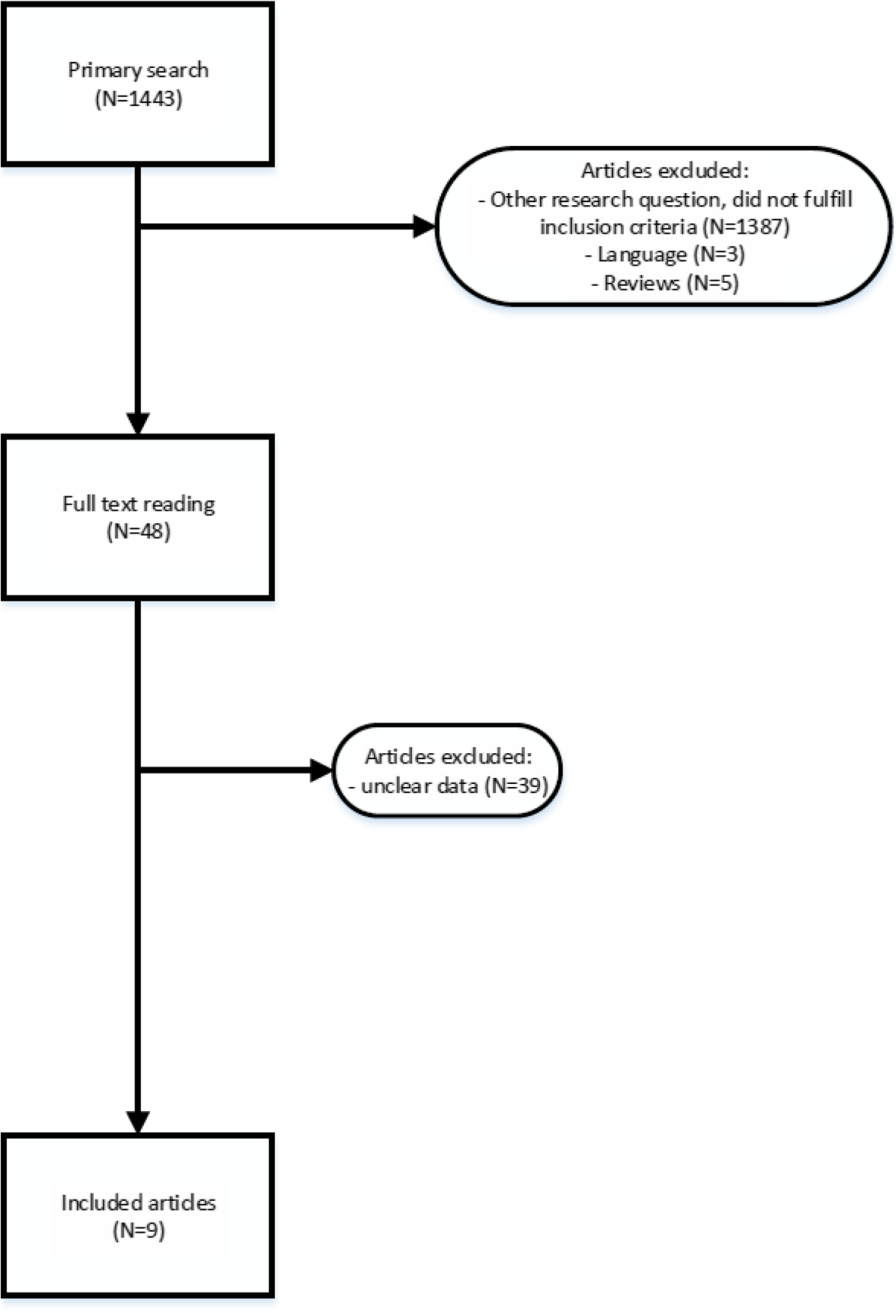
Inclusion flow chart

Figure 1 demonstrates the inclusion flow chart, and the characteristics of the included studies are shown in Table 1. The methodological quality of each included study as scored by our assessment tool is reported in Table 2.

**Table 1:** Characteristics of included articles

Year	First author	Disease	Disease Diagnosis	N total	survivors	Died/ lost	NS	Seizure Diagnosis	Normal	Adverse	Epilepsy	Design P/R	S/M
2000	C. Sreenan	Stroke	CT	46	46	0 / 0	42	Clinical and EEG	15	31	21	P	M
2007	M.R. Golomb	Stroke	CT/ MRI	64	61	0 / 3	48	EEG	–	–	28	P	S
2008	D.Ricci	Stroke	MRI	31	28	1 / 2	26	EEG	12	16	6	P	S
2010	H.J. Lee	Stroke	MRI	13^b^	7	0 / 0	5	EEG	4	3	0	R	M
2004	S.P. Miller	Asphyxia	ES EEG	68	60	8 / 0	29	Clinical mainly	46	14	–	P	S
2009	F. Pisani	Asphyxia	Sarnat Amiel –Tison Cer. U.S.	92^a^	57	0 / 0	18	Video EEG	47	10	3	R	S
2009	N. Al-Macki	Asphyxia	Sarnat EEG MRI	40	40	0 / 0	33	EEG	17	23	–	R	S
2009	H.C. Glass	Asphyxia	MRI	143	77	16 / 67	25	Video EEG	60	17	–	P	S
2013	L.A. Beslow	Hemorr.	CT MRI	73^c^	20	1 / 0	12	Clinical and EEG	16	1	1	P	M

Disease: brain injury type investigated in study; Disease Diagnose: diagnostic tool/measure/score assessing injury (Cer. US: cerebral ultrasound, MRI: magnetic resonance imaging): ES: encephalopathy score; N total: number of cases in study; Survivors: number of cases survived for follow up, NS: neonatal seizures number of cases; Adverse: adverse developmental outcome; Epilepsy: number of cases with late onset epilepsy; Design P/R: Prospective/Retrospective; S/M: Single-center/Multi-center study. ^a^92 newborns with asphyxia, only 57 infants developed clinical HIE and were followed. ^b^total number included 13 but 6 preterm infants. ^c^total included 73 cases but only 20 perinatal term cases

**Table 2:** Methodological quality of included studies

Year	First author	Disease	1 Selection 6 items	2 Diagnosis 3 items	3 Outcome 5 items	4 Flow/Timing 6 items	MQTotal 20 items
2000	C. Sreenan^41^	Stroke	5	3	3	5	16
2007	M.R. Golomb^22^	Stroke	6	3	1	5	15
2008	D.Ricci^37^	Stroke	6	3	4	6	19
2010	H.J. Lee^28^	Stroke	6	3	3	6	18
2004	S.P. Miller^31^	Asphyxia	6	1	5	6	18
2009	F. Pisani^35^	Asphyxia	5	3	3	6	17
2009	N. Al-Macki^5^	Asphyxia	6	3	3	6	18
2009	H.C. Glass^20^	Asphyxia	6	3	5	4	18
2013	L.A. Beslow^11^	Hemorrhage	6	3	4	6	19

Disease: brain injury type investigated in the study. The study score per main Quadas-2 item is demonstrated out of the maximum score per item. MQ total: total number of items scored positive for good methodological quality on Quadas-2 out of maximum of 20

Superscript numbers reflect the corresponding literature references

### Analyses of pooled data

For all cases with stroke or asphyxia combined the pooled risk ratio (RR) for adverse outcome when suffering neonatal seizures was 7.42 (3.84–14.34), I^2^ = 0%, Chi^2^ = 1.47.

In neonates with perinatal asphyxia suffering neonatal seizures the pooled RR for adverse outcome was 8.41 (4.07–17.39), I^2^ = 0%, Chi^2^ = 0.84.

In neonates with stroke suffering neonatal seizures the pooled RR for adverse outcome 4.95 (1.07–23.0), I^2^ = 0%, Chi^2^ = 0.21.

The pooled RR for development of late onset epilepsy could only be determined for infants suffering from stroke: 1.48 (0.82–2.68), I^2^ = 0%, Chi^2^ = 2,75.

For both the etiologies HIE and cerebral hemorrhage only one study could be included which evaluated the development of epilepsy in infants with and without neonatal seizures. In the study of Pisani and colleagues (*n* = 57) late onset epilepsy was found in three HIE patients of in total 18 newborns suffering neonatal seizures and in none of the patients without neonatal seizures (*n* = 39). In the study of Beslow and colleagues (*n* = 17), including patients with cerebral hemorrhage, late onset epilepsy was found in one of the patients suffering neonatal seizures (*n* = 12) and in none of the patients without neonatal seizures (*n* = 5).

## Discussion

To our knowledge this is the first systematic review and meta-analysis on the prognostic value of neonatal seizures for long term outcome in term newborns suffering from either perinatal asphyxia, stroke or cerebral hemorrhage.

Newborns suffering from hypoxic or vascular brain injury *and* seizures in the neonatal period may have an increased risk of adverse neurodevelopmental outcome compared with newborns with the same brain injury *without* neonatal seizures. This is in accordance with previous findings in the literature [2–4, 7–9].

The results of this meta-analysis do not provide evidence for the theory that the seizures play a causative pathogenic role. However, this theory is supported by findings from experimental studies demonstrating multiple adverse cerebral effects of neonatal seizures. One of these adverse effects is microglia activation, as indicated by the morphologic changes and rapid production of pro inflammatory cytokines after the onset of acute seizures [23]. Microglia participate in “synaptic stripping” by detaching presynaptic terminals from neurons thereby altering brain structure and possibly brain function. Another seizure induced adverse effect on the brain development is a loss of subplate neurons, which are critical for the development of normal maturation of cortical networks [24].

The neonatal period is characterized by synaptic plasticity and learning. A major factor in this enhanced synaptic plasticity is the predominance of excitation over inhibition, which unfortunately also increases susceptibility to neonatal seizures. Seizure induced changes in these signaling pathways such as the GABA-ergic system, and changes in Glutamate receptors may result in long term impairments of brain function [5, 25].

These mechanisms may all influence long term outcome in newborns.

Most infants with neonatal seizures were treated with one or more anti-epileptic drugs (AEDs). There are reports that the use of (multiple) AEDs in the neonatal period is associated with adverse neurodevelopmental outcome [26, 27]. Obviously, this effect is confounded by indication and could not be well assessed on the basis of these data.

Although there is evidence that neonatal seizures influence both structure and function of the developing central nervous system, thereby possibly lowering the threshold for the development of epilepsy, we were not able to perform any meta-analysis on the risk for developing epilepsy in infancy. Only two studies, including small numbers of patients, reported on this outcome. The development of post neonatal epilepsy following neonatal seizures and brain damage may also be more difficult to predict as the location and severity of cortical damage may influence this. A recent literature study by Pisani et al. [28] demonstrated that around 18% of newborns suffering from neonatal seizures (regardless of brain injury type) developed post-neonatal epilepsy. The onset of epilepsy was within the first year of life in almost 70% of cases. Uncertainty remains about the true incidence of post neonatal epilepsy after neonatal seizures due to the lack of well-designed population-based studies taking into account the type of brain injury.

We had hoped the large cohort studies on hypothermia would have contributed to answering our research question but unfortunately they did not fulfill our inclusion criteria as follow up criteria were not met and the incidence and treatment of seizures was not documented. Maybe an individual patient data analysis performed based on data from these large cohorts of affected newborns can answer our questions.

This study has some severe limitations that need to be discussed. First, the number of studies and patients included in this review was relatively small. Many studies had to be excluded because they included both newborns and older children or term and preterm newborns. Unfortunately, the data were not separately reported for the different age groups.

Second the mode of seizure - or epilepsy diagnosis was often not well defined and third the outcome in most studies was either not assessed in a blinded manner or outcome assessments were performed too early in life. This reduced the number of eligible studies greatly. Confirmation bias (a bias based on personal experience); unclear selection bias in the populations described; model under fitting (trying to explain a too complex disease entity or outcome by single determinants) and the presence of unknown cofounders may all have influenced our results.

However, taking all this into account, the data we reviewed give at least an indication that suffering neonatal seizures during neonatal stroke, brain hemorrhage or perinatal asphyxia may have an impact on outcome for the developing child.

For this reason, the results of our review need to be confirmed by large prospective cohort studies addressing these issues. Also a well-designed, multicenter registry prospectively collecting data on neonates with brain injury, including the presence or absence of neonatal seizures, is urgently needed. The diagnostic criteria and outcome parameters collected in such a database should be well-defined and uniformly assessed.

## Conclusions

The presence of neonatal seizures combined with vascular or hypoxic brain injury in term newborns may have an impact on the neurodevelopmental outcome of affected newborns.

With the currently available data from the literature we could not determine the risk ratio of developing post-neonatal epilepsy following neonatal seizures and brain injury in term infants. One or more large prospective cohort studies are needed to confirm or refute these findings.

## Additional files

Additional file 1: Search strategy. (DOCX 31 kb)

Additional file 2: Quadas Tool. (DOCX 21 kb)

Additional file 3: Data extraction form. (DOC 72 kb)

## Abbreviations

AED: antiepileptic drug
BSID: Bailey States of Infant Development test
CI: confidence Interval
GA: gestational age
GABA: Gamma Amino Butyric Acid
GMFS: Global Motor Functioning Scale
HIE: hypoxic ischemic encephalopathy
MOOSE: Meta-analysis of Observational Studies in Epidemiology
NICU: neonatal intensive care unit
PRISMA: Preferred Reporting Items for Systematic Reviews and Meta-Analyses
RR: risk ratio
