# Supplementary material for: A systematic review comparing neurodevelopmental outcome in term infants with hypoxic and vascular brain injury with and without seizures
Source: BMC Pediatr. 2018 May 2;18:147. doi: 10.1186/s12887-018-1116-9 (PMC5930747; doi:10.1186/s12887-018-1116-9)
Supplement: Supplementary file 1 — Search strategy. (DOCX 31 kb) [file 12887_2018_1116_MOESM1_ESM.docx]

## Additional file 1 search data meneer leenders

**20JAN2015**

**MEDLINE**

1. exp Epilepsy/ or epileps*.tw.

2. exp Intracranial Hemorrhages/ or (intracranial adj3 hemorrhag*).tw.

3. Asphyxia Neonatorum/ or (asphyxia adj3 neonatorum).tw.

4. exp Meningitis/ or meningitis.tw.

5. exp Brain Infarction/ or (stroke or (brain adj3 infarct*)).tw.

6. exp Encephalitis/ or encephalit*.tw.

7. or/2-6 ["hersenproblemen"]

8. 1 and 7

9. Infant, Newborn/ or (neonat* or newborm* or aterm or a term).tw.

10. 8 and 9

11. exp mortality/

12. exp Nervous System/di, gd, me, pa, pp [Diagnosis, Growth & Development, Metabolism, Pathology, Physiopathology]

13. exp mental disorders diagnosed in childhood/

14. exp Child Development/

15. exp Nervous System Diseases/

16. exp Brain Damage, Chronic/

17. (neurological adj3 (outcome or sequel*)).tw.

18. or/11-17 [neurologic outcome]

19. 10 and 18

20. exp epidemiologic studies/ or (cohort* or retrospective or prospective or follow up or case control or longitudinal).tw.

21. exp prognosis/ or prognos*.tw. or predict*.tw.

22. 20 or 21

23. 10 and 22

24. infant/ or schoolchild/ or (infan* or toddler* or schoolchild* or school child*).tw.

25. 8 and 24

26. 10 and 24

27. 23 or 26

28. 19 and 20

29. (epileps* adj5 (newborn* or neonat* or term or termborn)).tw.

30. 20 and 29

31. 7 and 29

32. 30 or 31

33. 26 and 22

34. 19 or 23

35. 24 and 34

**20jan2015**

**EMBASE**

1. exp Epilepsy/ or epileps*.tw.

2. exp Intracranial Hemorrhages/ or (intracranial adj3 hemorrhag*).tw.

3. Asphyxia Neonatorum/ or (asphyxia adj3 neonatorum).tw.

4. exp Meningitis/ or meningitis.tw.

5. exp Brain Infarction/ or (stroke or (brain adj3 infarct*)).tw.

6. exp Encephalitis/ or encephalit*.tw.

7. or/2-6 ["hersenproblemen"]

8. 1 and 7

9. Infant, Newborn/ or (neonat* or newborm* or aterm or a term).tw.

10. 8 and 9

11. exp mortality/

12. exp Nervous System/

13. exp mental disorders diagnosed in childhood/

14. exp Child Development/

15. exp Nervous System Diseases/

16. exp Brain Damage, Chronic/

17. (neurological adj3 (outcome or sequel*)).tw.

18. or/11-17 [neurologic outcome]

19. 10 and 18

20. exp epidemiologic studies/ or (cohort* or retrospective or prospective or follow up or case control or longitudinal).tw.

21. exp prognosis/ or prognos*.tw. or predict*.tw.

22. 20 or 21

23. 10 and 22

24. infant/ or schoolchild/ or (infan* or toddler* or schoolchild* or school child*).tw.

25. 8 and 24

26. 10 and 24

27. 23 or 26

28. 19 and 20

29. (epileps* adj5 (newborn* or neonat* or term or termborn)).tw.

30. 20 and 29

31. 7 and 29

32. 30 or 31

33. 26 and 22

34. 19 or 23

35. 24 and 34
