# Supplementary material for: A systematic review comparing neurodevelopmental outcome in term infants with hypoxic and vascular brain injury with and without seizures
Source: BMC Pediatr. 2018 May 2;18:147. doi: 10.1186/s12887-018-1116-9 (PMC5930747; doi:10.1186/s12887-018-1116-9)
Supplement: Supplementary file 2 — Quadas Tool. (DOCX 21 kb) [file 12887_2018_1116_MOESM2_ESM.docx]

**Assessing methodological quality: Risk of Bias and Applicability Judgement**

*Based on QUADAS-2*

Rater:

Author:

Date of publication:

Endnote Number:

*Patient Selection*

- Describe methods of patient selection

- Describe included patients (previous testing, presentation, intended use of EEG)
- Was a consecutive or random sample of patients enrolled? yes/no/unclear
- Did the study avoid inappropriate exclusion? yes/no/unclear
- Could the selection of patients have introduced bias? Yes/ no/ unclear
- Are there concerns that the included patients do not match the review question? Yes/ no/ unclear

*Diagnosis Epi9lepsyEEG*

- Describe how the EEG was conducted and interpreted

- Could the conduct or interpretation of the EEG have introduced bias? Yes/ no/ unclear
- Are there concerns that the EEG, its conduct or its interpretation differ

from the review question? Yes/ no/ unclear

*Reference standardOutcome*

- Describe the outcome test and how it was conducted and interpreted
- Is the outcome test likely to correctly classify the target condition? yes/no/unclear
- Were the outcome test results interpreted without knowledge of either the EEG or clinical course yes/no/unclear
- Could the outcome test, its conduct, or its interpretation have introduced bias? Yes/ no/ unclear
- Are there concerns that the target condition as defined by the follow-up does

not match the review question? Yes/ no/ unclear

*Flow and Timing*

- Describe any patients who did not receive the EEG or predictive test (loss-to-follow-up) or who were excluded from the 2x2 table (refer to flow diagram)
- Describe the interval and any interventions between EEG and the predictive test
- Did all patients receive a follow-up measurement? yes/no/unclear
  - Percentage of loss-to follow up ____%
- Did all patients receive the same follow-up measurement? yes/no/unclear
- Were all patients included in the analysis? yes/no/unclear
- Could the patients flow have introduced bias? Yes/ no/ unclear
