# Supplementary material for: A systematic review comparing neurodevelopmental outcome in term infants with hypoxic and vascular brain injury with and without seizures
Source: BMC Pediatr. 2018 May 2;18:147. doi: 10.1186/s12887-018-1116-9 (PMC5930747; doi:10.1186/s12887-018-1116-9)
Supplement: Supplementary file 3 — Data extraction form. (DOC 72 kb) [file 12887_2018_1116_MOESM3_ESM.doc]

**Standardized data extraction form**

Rater:

Author:

Date of publication:

Endnote number:

**Characteristics of the study**

***Participants***

Total number of cases

Number of New-borns male: female:

Number of new-borns with stroke?

Number of new-borns with perinatal asphyxia?

Number of new-borns with cerebral bleeding?

Number of new-borns with CSN infections?

Number of new-borns with neonatal seizures?

Number of cases treated?

Number of cases with > 2 AEDs?

Number of cases with status?

***Way of diagnostic epilepsy***

Age at time of diagnosis (in days)?

EEG confirmed diagnose?

Other means of diagnosis?

- only clinical?
- aEEG?

***Study design***

Prospective/Retrospective

Single/Multi centre

***Test at follow-up***

Age at follow-up ?

Type of tests used?

Which areas do these tests cover?

□ Neurologic performance

□ Behaviour

□ Cognitive

□ Somatic

**Results of the study**

***Main finding (related to prognostic factor)***

***2x2 table***

| ***Patients with stroke*** | ***Died*** | ***Survived*** | ***Total*** |
| --- | --- | --- | --- |
| ***Neonatal seizures Yes*** |  |  |  |
| ***Neonatal seizures No*** |  |  |  |
| ***Total*** |  |  |  |

| ***Patients with stroke*** | ***Post-neonatal epilepsy*** | ***No epilepsy*** | ***Total*** |
| --- | --- | --- | --- |
| ***Neonatal seizures Yes*** |  |  |  |
| ***Neonatal seizures No*** |  |  |  |
| ***Total*** |  |  |  |

| ***Patients with stroke*** | ***Psychomotor retardation**** | ***Normal*** | ***Total*** |
| --- | --- | --- | --- |
| ***Neonatal seizures Yes*** |  |  |  |
| ***Neonatal seizures No*** |  |  |  |
| ***Total*** |  |  |  |

*including CP

***2x2table***

| ***Patients with perinatal asphyxia*** | ***Died*** | ***Survived*** | ***Total*** |
| --- | --- | --- | --- |
| ***Neonatal seizures Yes*** |  |  |  |
| ***Neonatal seizures No*** |  |  |  |
| ***Total*** |  |  |  |

| ***Patients with perinatal asphyxia*** | ***Post-neonatal epilepsy*** | ***NO epilepsy*** | ***Total*** |
| --- | --- | --- | --- |
| ***Neonatal seizures Yes*** |  |  |  |
| ***Neonatal seizures No*** |  |  |  |
| ***Total*** |  |  |  |

| ***Patients with perinatal asphyxia*** | ***Psychomotor retardation*** | ***Normal*** |  |
| --- | --- | --- | --- |
| ***Neonatal seizures Yes*** |  |  |  |
| ***Neonatal seizures No*** |  |  |  |
| ***Total*** |  |  |  |

2x2 Table

| ***Patients with cerebral haemorrhage*** | ***Died*** | ***Survived*** | ***Total*** |
| --- | --- | --- | --- |
| ***Neonatal seizures Yes*** |  |  |  |
| ***Neonatal seizures No*** |  |  |  |
| ***Total*** |  |  |  |

| ***Patients with cerebral haemorrhage*** | ***Post-neonatal epilepsy*** | ***No epilepsy*** | ***Total*** |
| --- | --- | --- | --- |
| ***Neonatal seizures Yes*** |  |  |  |
| ***Neonatal seizures No*** |  |  |  |
| ***Total*** |  |  |  |

| ***Patients with cerebral haemorrhage*** | ***Psychomotor retardation*** | ***Normal*** | ***Total*** |
| --- | --- | --- | --- |
| ***Neonatal seizures Yes*** |  |  |  |
| ***Neonatal seizures No*** |  |  |  |
| ***Total*** |  |  |  |

**Combined Tables:**

| ***Combined table***  ***STROKE*** | ***Adverse*** | ***Normal*** |
| --- | --- | --- |
| ***Neonatal seizures Yes*** |  |  |
| ***Neonatal seizures No*** |  |  |

| ***Combined table***  ***Asphyxia*** | ***Adverse*** | ***Normal*** |
| --- | --- | --- |
| ***Neonatal seizures Yes*** |  |  |
| ***Neonatal seizures No*** |  |  |

| ***Combined table***  ***Cerebral haemorrhage*** | ***Adverse*** | ***Normal*** |
| --- | --- | --- |
| ***Neonatal seizures Yes*** |  |  |
| ***Neonatal seizures No*** |  |  |
